# Highly efficient evaporative cooling by all-day water evaporation using hierarchically porous biomass

**DOI:** 10.1038/s41598-021-96303-w

**Published:** 2021-08-19

**Authors:** Jihun Choi, Hansol Lee, Bokyeong Sohn, Minjae Song, Sangmin Jeon

**Affiliations:** Department of Chemical Engineering, Pohang University of Science and Technology (POSTECH), Pohang, Gyeongbuk 37673 Republic of Korea

**Keywords:** Energy science and technology, Materials science

## Abstract

We developed a 3D solar steam generator with the highest evaporation rate reported so far using a carbonized luffa sponge (CLS). The luffa sponge consisted of entangled fibers with a hierarchically porous structure; macropores between fibers, micro-sized pores in the fiber-thickness direction, and microchannels in the fiber-length direction. This structure remained after carbonization and played an important role in water transport. When the CLS was placed in the water, the microchannels in the fiber-length direction transported water to the top surface of the CLS by capillary action, and the micro-sized pores in the fiber-thickness direction delivered water to the entire fiber surface. The water evaporation rate under 1-sun illumination was 3.7 kg/m^2^/h, which increased to 14.5 kg/m^2^/h under 2 m/s wind that corresponded to the highest evaporation rate ever reported under the same condition. The high evaporation performance of the CLS was attributed to its hierarchically porous structure. In addition, it was found that the air temperature dropped by 3.6 °C when the wind passed through the CLS because of the absorption of the latent heat of vaporization. The heat absorbed by the CLS during water evaporation was calculated to be 9.7 kW/m^2^ under 1-sun illumination and 2 m/s wind, which was 10 times higher than the solar energy irradiated on the same area (1 kW/m^2^).

## Introduction

Urban population is rapidly growing, and by 2050, 68% of the world population is expected to live in cities^1^. Urbanization has caused many unexpected problems. One of these problems is the urban-heat-island (UHI) effect that makes urban areas warmer than the surrounding rural areas^2,3^. Evaporative cooling in an urban area, which lowers environmental temperature by absorbing the latent heat of vaporization during water evaporation, is an economic and effective strategy for mitigating the UHI effect^4–6^. Water evaporation can be promoted by increasing blue spaces such as lakes and green spaces such as trees. However, blue spaces induce night-time warming effects by releasing stored heat at night, and green spaces require constant care and stop transpiration at night^7,8^. Therefore, a new strategy needs to be developed to realize highly efficient all-day water evaporation.

Solar steam generation (SSG) is a promising and sustainable alternative to natural trees because this technology utilizes solar energy for water evaporation^9–13^ without the problems on pests and diseases. The high efficiency of SSGs for water evaporation comes from heat localization in solid–liquid–air interfaces that suppresses heat loss to bulk water^14–17^. However, maximum evaporation rate using conventional SSGs with 2D structures was found to be only 1.5 kg/m^2^/h because it was limited by the amount of absorbed solar energy per area^18,19^. The maximum value can be exceeded by lowering the enthalpy of vaporization of water^20,21^ or adopting a 3D-structured SSG^22^. In particular, the use of 3D SSG can greatly improve the evaporation performance because it utilizes not only solar energy but also environmental energy such as wind more efficiently^19,23,24^.

Water evaporation in a 3D SSG is divided into solar and dark evaporation according to energy sources^18,25^. Solar evaporation performance of a 3D SSG is governed by efficiencies of solar energy absorbance, thermal insulation, and water transport^26,27^. Structure and material of an SSG must be designed to ensure high solar energy absorbance to evaporate water^28,29^, high thermal insulation to prevent the dissipation of absorbed energy to bulk water^15,30^, and sufficient water transport to the surface of a 3D SSG where evaporation takes place^31–33^. When fabricating 3D SSGs using natural materials, natural degradation issues must also be considered^34^.

In contrast to solar evaporation, dark evaporation utilizes environmental energy absorbed from its surroundings for water evaporation^25,35^. Dark evaporation performance of 3D SSG is mainly determined by the effective surface area in contact with a convective airflow^24,36^. To increase effective surface area, a 3D SSG must have a hierarchically porous structure consisting of micro-sized pores and macropores^37^. If the 3D SSG only contains micro-sized pores inside, the apparent surface area and water transport can be improved, but the water-filled micro-sized pores prevent airflow from contacting the inner surface of the SSG, reducing the effective surface area and causing water evaporation to occur only from the outer surface. In contrast, if the 3D SSG has only macropores that allow airflow to contact the inner surface, weak capillary forces prevent water from moving to higher elevations, limiting the height of the SSG and reducing the effective surface area.

Artificial 3D SSGs with various structures such as column^19,23,38^, cup^39^, cone^40^, fin^18^, and hemispheric shapes^41^ have been developed, but a hierarchically porous structure has not been implemented because of fabrication difficulties. In contrast, plants in nature have evolved to possess hierarchically porous channel structures for efficient water transport, transpiration, and sunlight absorbance^42–45^. In particular, luffa sponge (LS) has been used as a natural kitchen sponge owing to its unique porous structure and high water-uptake performance^46,47^. LS is composed of entangled luffa fibers (LFs), and the macropores among the LFs allow airflow to pass through the LS. LF consists of a bundle of microchannels^48^ connected through lateral micro-sized pores, which enable water transport to a high elevation and a LF surface.

In this study, we developed a 3D SSG using a carbonized LS (CLS) that was obtained by the carbonization of LS. The unique hierarchically porous structure of LS remained in the CLS, which allowed solar evaporation under daylight illumination and dark evaporation throughout a day. The dark evaporation rate was 1.4 kg/m^2^/h, which was close to the best result of conventional 2D SSGs under 1-sun illumination. The evaporation rate significantly increased with the presence of airflow by facilitating water evaporation. The water evaporation rate under 1-sun illumination and wind speed of 2 m/s was 14.5 kg/m^2^/h, which corresponded to the highest evaporation rate ever reported under the same condition. In addition, the heat absorbed by the CLS during water evaporation was found to be 10 times greater than solar energy irradiated on the same area, indicating that CLS can be used to mitigate the UHI effect by promoting evaporative cooling.

## Methods

### Materials

Dried LS (DLS) was purchased from Yojyo Factory (Yeoju, Korea). Melamine foam (MF) and polyurethane foam (PUF) were purchased from BASF (Ludwigshafen, Germany) and Clean Life (Hanam, Korea), respectively. Deionized water (18.3 MΩ cm) was obtained from a reverse osmosis water system (Human Science, Korea).

### Carbonization

To obtain CLS, the DLS was compressed to form a cylindrical shape and carbonized in a tube furnace (Daemyoung Enterprise, Korea) at 450 °C for 2 h under nitrogen atmosphere. Carbonized MF (CMF) was obtained by cutting the MF into a cylindrical shape and carbonizing it in a tube furnace under the same condition. PUF did not need to be carbonized because it was basically black. The diameter and height of the resulting samples were 25 and 70 mm, respectively.

### Characterization

Morphology of each sample was analyzed using a scanning electron microscope (SEM). The average diameter of microchannels was calculated using ImageJ (NIH). Fourier transform infrared (FT-IR) spectra were obtained using an FT-IR spectrometer (FT/IR-4600, JASCO, Japan). Diffuse reflectance and transmittance spectra were measured using a UV–Vis–NIR spectrophotometer (Cary 5000, Varian, CA, USA) equipped with an integrated sphere for reflected-light collection.

### Evaporation performance

After placing the CLS in a 60 mL water container, mass change in the water during evaporation was measured using an electronic balance, and surface temperature was monitored using an IR camera (FLIR A315, FLIR Systems, Inc., OR, USA). A solar simulator (PEC-L11, Peccell Technologies, Japan) was used for uniform illumination and the light intensity was measured using an optical power meter (1919-R, Newport, CA, USA). Airflow was generated using an electric fan with a blade length of 3.5 cm, and wind speed was measured using an anemometer (GM816, BENETECH, China). Evaporation rate was calculated by dividing the change in mass of water for 1 h by the area occupied by each sample. The air temperature at the inlet and outlet of the CLS was measured using thermistors (TSP-TH, Thorlabs, NJ, USA) placed with a 2 mm gap between the thermistor and the CLS. All measurements were conducted at 23 ± 1 °C and a relative humidity of 39 ± 5%.

## Results and discussion

**Figure 1:**
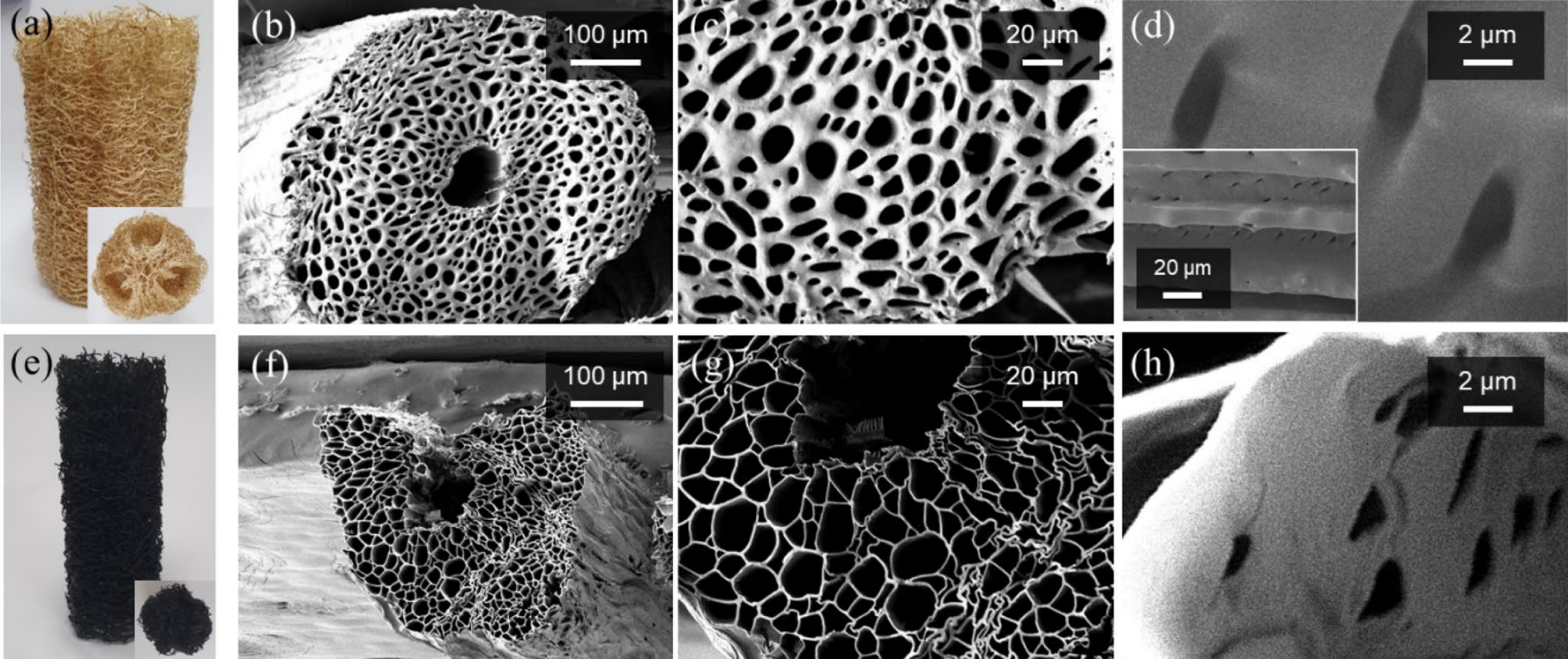
(**a**) Photograph of the DLS. The inset shows the cross-sectional image. (**b**) Cross-sectional SEM image of the LF and (**c**) its magnified image. (**d**) Cross-sectional SEM image of the LF in the fiber-length direction. The inset image at low magnification shows that micro-sized pores exist on the microchannel surface. (**e**) Photograph of CLS. The inset shows the cross-sectional image. (**f**) Cross-sectional-view SEM image of a CLS fiber and (**g**) its magnified image. (**h**) Cross-sectional SEM image of a CLS fiber in the fiber-length direction.

Figure 1a and its inset show the side- and cross-sectional-view photographs of the 100-mm-diameter DLS, respectively. The three large voids inside the DLS indicated the locations occupied by the seeds. The inner part of the DLS was removed, and outer part was compressed to obtain a cylindrical shape. The DLS diameter decreased from 100 to 40 mm after compression (not shown here). The millimeter-sized macropores between the LFs allowed wind to pass through the DLS. Figure 1b,c show the cross-sectional SEM images of the DLS fiber at different magnification scales. A number of microchannels with an average diameter of 11 μm surrounded a large microchannel with a diameter of ~ 70 μm at the center of the DLS fiber. These microchannels transported water to the upper part of the DLS fiber through capillary action. Figure 1d and its inset show the micro-sized pores on the microchannel surface under different magnifications. The micro-sized pores connected a microchannel to the adjacent microchannels and delivered water to the outer surface of the LF.

The compressed DLS was carbonized to convert it into a photothermal carbonaceous material under nitrogen atmosphere at 450 °C for 2 h. Figure 1e shows that the diameter decreased from 40 to 25 mm after carbonization, and CLS color turned from bright brown to black. The millimeter-sized macropores between the entangled fibers remained after carbonization. Figure 1f,g show the cross-sectional SEM images of the CLS fiber under different magnification scales. Figure 1h shows the micro-sized pores on the microchannel surface of the CLS fiber. No significant changes were observed in the average diameters of the microchannels and micro-sized pores between the DLS and CLS, indicating that the porous structure was maintained even after carbonization.

**Figure 2:**
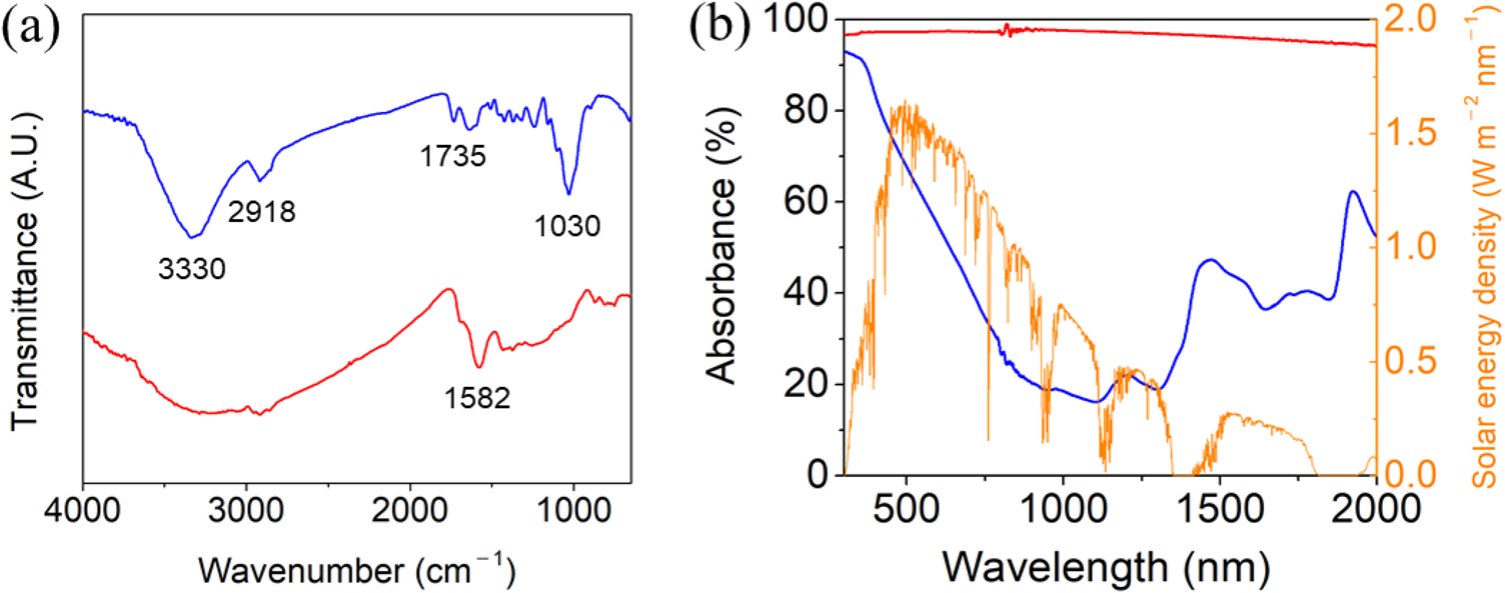
(**a**) FT-IR spectra and (**b**) light adsorption spectra of the DLS (blue) and CLS (red). The light absorbance is obtained by normalizing with AM 1.5G spectrum (orange).

The changes in the functional groups after carbonization were investigated through an FT-IR analysis (Fig. 2a). The peaks corresponding to the O–H stretching vibration (3330 cm^−1^) and –OH in-plane bending vibration (1030 cm^−1^) disappeared after carbonization, indicating that a dehydration reaction occurred. The decrease in the peak at 1735 cm^−1^ corresponded to a deoxidation reaction. The increase in the peak at 1582 cm^−1^ (C=C bond) and decrease at 2918 cm^−1^ (C–H bond) indicated the formation of aromatic or unsaturated carbon-to-carbon bonding via dehydrogenation. The dehydration, deoxidation, and dehydrogenation reactions during carbonization increased the proportion of atomic carbon in the CLS, resulting in the increase in light absorption^49–52^. Figure 2b shows that the light absorbance of the CLS sample was 97% in the range of 300–2000 nm, whereas, that of the DLS was only 38%.

**Figure 3:**
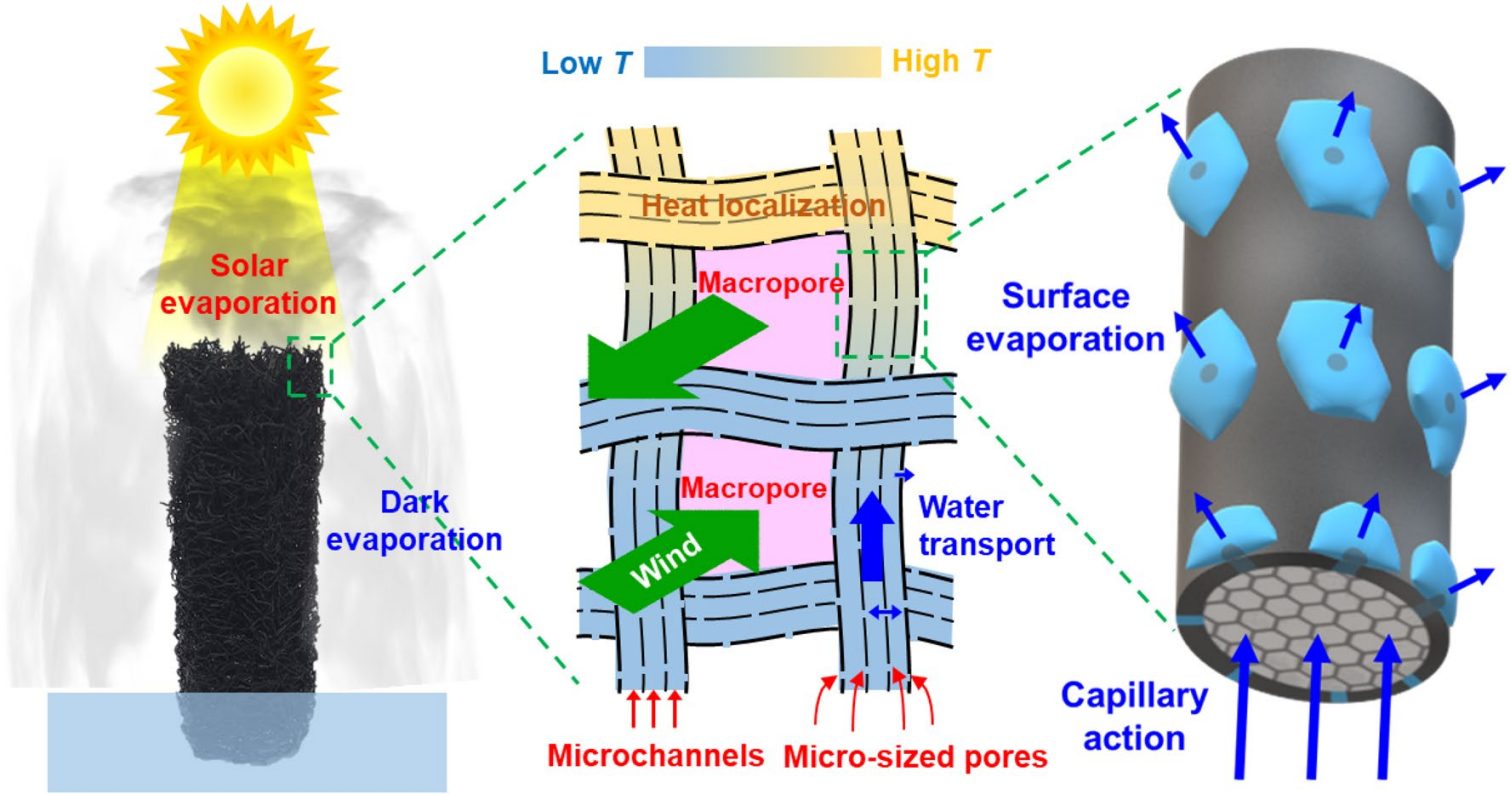
Schematic of the solar and dark evaporation occurring in the CLS. Solar evaporation occurs from the top surface of the CLS, and dark evaporation occurs from the entire surface of the CLS regardless of the illumination. The macropores between the CLS fibers allow wind to pass through the CLS, promoting dark evaporation. The micro-sized pores connected with adjacent microchannels transfer water to the outer surface of the CLS fiber. The microchannels promote water transport to the top of the CLS fibers.

Figure 3 shows the schematic of the water evaporation that occurred in the CLS. Solar evaporation occurred from the top surface of the CLS wherein water was delivered through the microchannels in the fiber-length direction. The maximum height of water that was transported through the microchannels (*H*) can be calculated by^53^

1 $$H=\frac{2\gamma\, {\cos}\theta }{\rho gR},$$

where *γ* and *ρ* are the surface tension and density of water, respectively, *θ* is water contact angle on the microchannel surface, *g* is gravitational acceleration, and *R* is microchannel radius (5.5 μm). Assuming that *θ* is 30° for calculation (a conservative approximation to hydrophilic CLS), the theoretical maximum value of *H* corresponds to 2.3 m. Dark evaporation occurred from the entire surface irrespective of illumination, and water was transported from the microchannels to the surface through the micro-sized pores. The macropores between the CLS fibers suppressed the conduction of absorbed heat from the top surface to the lower part of the CLS and allowed wind to pass through the CLS, promoting both solar and dark evaporation.

**Figure 4:**
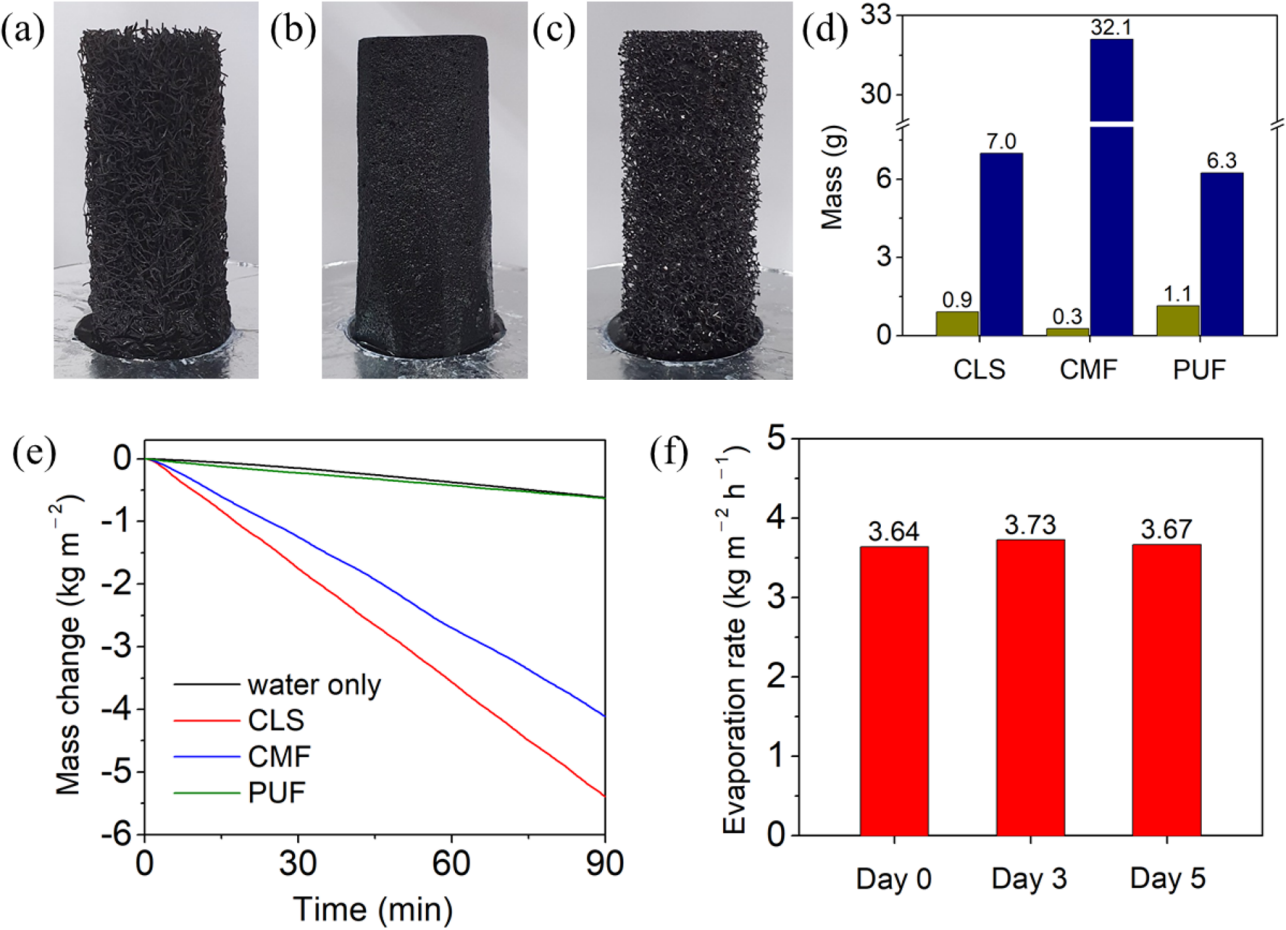
Photograph of (**a**) CLS, (**b**) CMF, and (**c**) PUF. The height of each sample above the water surface is 60 mm. (**d**) Dry masses (dark yellow) and wet masses (dark blue) of the CLS, CMF, and PUF. (**e**) Evaporation-induced mass change of water with each sample under 1-sun illumination. (**f**) Variation in the evaporation rate of water with CLS under 1-sun illumination over 5 days.

To investigate the influence of the pore structures on the water evaporation performance, the evaporation rates of the CLS, CMF, and PUF with different pore structures were compared. Figure 4a–c show the photographs of CLS, CMF, and PUF, respectively, with the same dimension. CLS and PUF were composed of loosely structured fibers with a few millimeters of macropores between the fibers, and CMF were composed of densely structured fibers with ~ 100 μm of macropores. Note that microchannels existed in the fiber-length direction inside the CLS fibers, but no microchannels were present inside CMF and PUF fibers. Each sample was placed in a water container, and the height of the sample above the water surface was 60 mm. Figure 4d shows the comparison of the dry and wet masses of each sample before and after water absorption. The dry mass of the CMF (0.3 g) was lower than that of the CLS (0.9 g) and PUF (1.1 g), indicating that the density of the CMF fibers was lower than that of the CLS and PUF fibers. However, the wet mass of the CMF was more than that of the CLS and PUF because of the larger capillary force of the CMF due to its smaller macropores than that of the CLS and PUF. Considering that the apparent volume of the CMF was 34.4 cm^3^ and the amount of water absorbed by the 0.3 g CMF was 31.8 g, the CMF macropores were almost completely filled with water. In contrast, the percentages of water filled in the CLS and PUF were 18% and 15%, respectively, indicating that the capillary forces induced by the millimeter-sized macropores in the CLS and PUF were insufficient to transport water to the upper parts.

Figure 4e shows the evaporation-induced mass changes of water with CLS, CMF, or PUF. The linear decrease in the mass indicated that the water evaporation was in a steady state during the measurements. The steady-state evaporation rate was calculated by dividing the mass change by the measurement time (1 h) and area (4.9 × 10^−4^ m^2^) occupied by each sample. Under 1-sun illumination, the CLS exhibited the highest evaporation rate (3.7 kg/m^2^/h), followed by the CMF (2.9 kg/m^2^/h) and PUF (0.4 kg/m^2^/h), which was contrary to the expectation that CMF would exhibit the highest evaporation rate because it absorbed the maximum amount of water. However, CMF (where the water completely filled the inner macropores) could only utilize the outer surface for water evaporation, resulting in poor dark evaporation performance. In contrast, the macropores in the CLS allowed water evaporation to occur from the internal surface, which increased the effective surface area for water evaporation. The dark evaporation rate of the CLS without illumination (1.4 kg/m^2^/h) was higher than that of the CMF (1.1 kg/m^2^/h), which confirmed that the CLS had a larger effective surface area than the CMF (Fig. S1 in the Supplementary Information).

In contrast to the CMF, PUF possessed macropores similar in size to the CLS; however, its water evaporation performance was the worst compared with the CMF and CLS. The poor performance of the PUF was attributed to the absence of microchannels inside the PUF fibers. Because the maximum height of water that can be transported through the 2-mm-diameter macropores was only 15 mm, water could only fill the lower part of PUF, which prevented solar evaporation from occurring at the top surface and reduced the effective surface area for dark evaporation. To deliver water to the top surface, the sample must contain microchannels for sufficient capillary action. The results obtained from the CMF and PUF demonstrated that both the macropores and microchannels played an important role in increasing the water evaporation performance.

To investigate whether the CLS with both macropores and microchannels could be used for long-term operation, the evaporation rate of water with CLS under 1-sun illumination was monitored over 5 days. Figure 4f shows that the evaporation rate was almost constant for 5 days of water evaporation, confirming the possibility of CLS for long-term operation. Note that the water evaporation rate of CLS (3.7 kg/m^2^/h) under 1-sun illumination corresponded to the highest evaporation rate ever reported under the same condition. Table 1 lists the comparison of the evaporation rates of water using various 3D SSGs.

**Table 1:** Evaporation rates of water using various 3D SSGs.

Material	Wind (m/s)	Evaporation rate (kg/m^2^/h)	References
Carbon black-coated cotton core	0	1.62	^19^
Metal oxide-coated glass fiber membrane cup		2.04	^39^
rGO-coated paper fin		2.94	^18^
Carbon-PMMA hemisphere		1.33	^41^
3D-printed acrylic cone		2.63	^40^
CNT coated corn stalk		2.48	^33^
Carbonized bamboo		3.13	^35^
Graphene-rice straw aerogel		2.25	^38^
Carbon snake	0	2.5	^24^
	2	7.8	
	6	10.9	
Carbonized luffa sponge	0	3.7	This work
	1	9.6	
	2	14.5	

**Figure 5:**
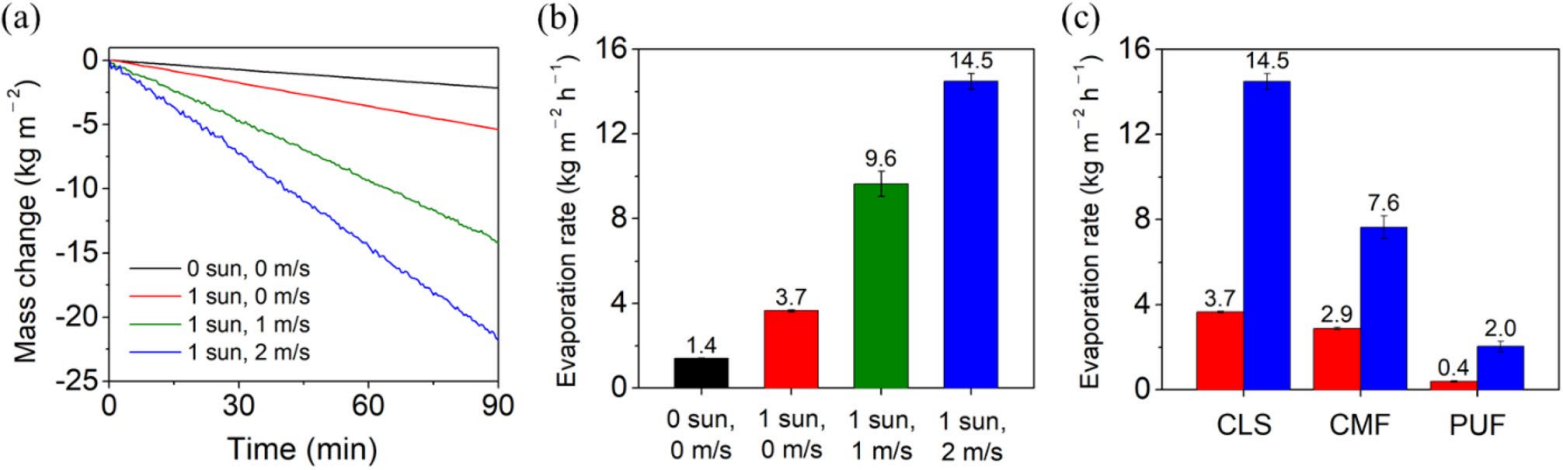
(**a**) Time-dependent changes in the mass of water under various conditions. (**b**) Evaporation rates of water with CLS under various conditions. The error bar represents the standard deviation obtained using three independent measurements. (**c**) Evaporation rates of water with CLS, CMF, and PUF under 1-sun illumination without wind (red) and with 2 m/s wind (blue).

The water evaporation rate can be further increased in the presence of wind. Figure 5a,b show the time-dependent changes in the mass of water with CLS under various conditions and the corresponding evaporation rates, respectively. The evaporation rate of CLS under 1-sun illumination without wind (3.7 kg/m^2^/h) increased to 9.6 kg/m^2^/h in the presence of 1 m/s wind, which further increased to 14.5 kg/m^2^/h when the wind speed increased to 2 m/s, which was the highest evaporation rate that has been reported so far. The difference in the evaporation rate between the presence and absence of 1-sun illumination was 2.3 kg/m^2^/h whereas, that between the presence and absence of 2 m/s wind under 1-sun illumination was 10.8 kg/m^2^/h. The increase in the water evaporation rate by the 2 m/s wind was approximately five times larger than that by 1-sun illumination. Considering that the maximum change in the illumination in nature is 1-sun but the maximum change in the wind speed is far greater than 2 m/s (a typhoon means a wind speed that exceeds 33 m/s), wind (i.e., dark evaporation) affects the water evaporation performance of 3D SSG much more significantly than solar illumination (i.e., solar evaporation). The water evaporation rate with CLS in the absence of wind and illumination was 1.4 kg/m^2^/h (Fig. S2 in the Supplementary Information), similar to the highest evaporation rate with conventional 2D SSGs under 1-sun illumination^18,19^. In addition, the evaporation rate with CLS (1.4 kg/m^2^/h) was seven times higher than that without CLS (0.2 kg/m^2^/h). Figure 5c shows the evaporation rates of water with CLS, CMF, or PUF under 1-sun illumination in the presence and absence of 2 m/s wind. The highest evaporation rate was observed for CLS, confirming that the 3D hierarchically porous structure of the CLS played a key role in increasing the evaporation rate. Note that the evaporation performance does not increase linearly as the number of CLS increases if the access of airflow through CLSs is not taken into account (Fig. S3 in the Supplementary Information).

**Figure 6:**
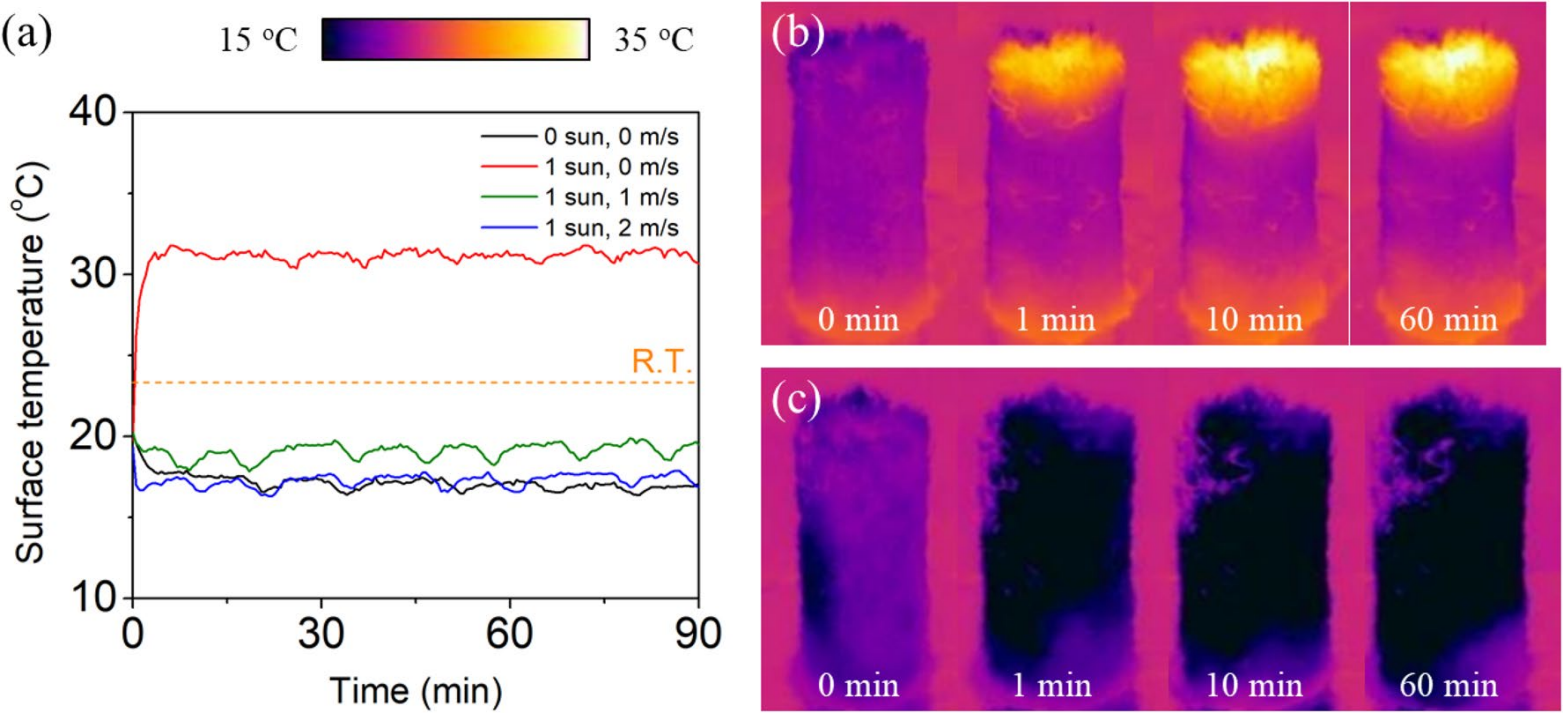
(**a**) Time-dependent changes in the average temperature at the top surface of CLS under various conditions. Time-lapse IR images of the CLS (**b**) under 1-sun illumination and no wind, (**c**) under 1-sun illumination and 2 m/s wind.

Figure 6a shows the time-dependent changes in the average temperature at the top surface of the CLS during water evaporation under various conditions. When the CLS was irradiated with sunlight and without wind, the surface temperature sharply increased, and it was saturated at 31 °C because of the equilibrium between the solar energy absorption and latent heat of vaporization. In the presence of 1 m/s wind, the top surface temperature decreased to 19 °C, which was lower than room temperature. A further decrease in surface temperature to 17 °C was observed under a 2 m/s wind. A similar surface temperature was observed in the absence of illumination and wind, confirming the importance of the dark evaporation. The surface temperature below room temperature indicated that CLS could absorb the energy from bulk water or the surrounding atmosphere^25,54^. Note that the low surface temperature reduced the radiation loss that was the dominant energy loss during solar evaporation^31,55^.

A decrease in the temperature of the CLS was observed not only on the top surface but also on the side surface. Figure 6b shows time-lapse IR images of the CLS under 1-sun illumination and no wind. The top surface temperature was higher than room temperature owing to solar irradiation, whereas the temperature at the water interface was similar to room temperature. However, the temperature in the middle part of the CLS was lower than room temperature, indicating that the dark evaporation from the middle part reduced the surface temperature by absorbing the latent heat of vaporization. The decrease in the surface temperature was highly apparent under 2 m/s wind (Fig. 6c). Because of the highly active water evaporation, the surface temperature of the entire CLS was substantially lower than room temperature.

The amount of heat absorbed by the CLS during water evaporation (Δ*Q*) can be calculated by

2 $$\Delta Q=\dot{m}{h}_{LV},$$

where $\dot{m}$ is the evaporation rate, and *h*_*LV*_ is the total enthalpy change for the liquid–vapor phase transition (2415 kJ/kg). The heat absorbed by the CLS during water evaporation was calculated to be 9.7 kW/m^2^ under 1-sun illumination at 2 m/s wind, which was ~ 10 times greater than the solar energy irradiated on the same area (1 kW/m^2^). This result implied that CLS could cool a surrounding area 10 times larger than that it occupies.

**Figure 7:**
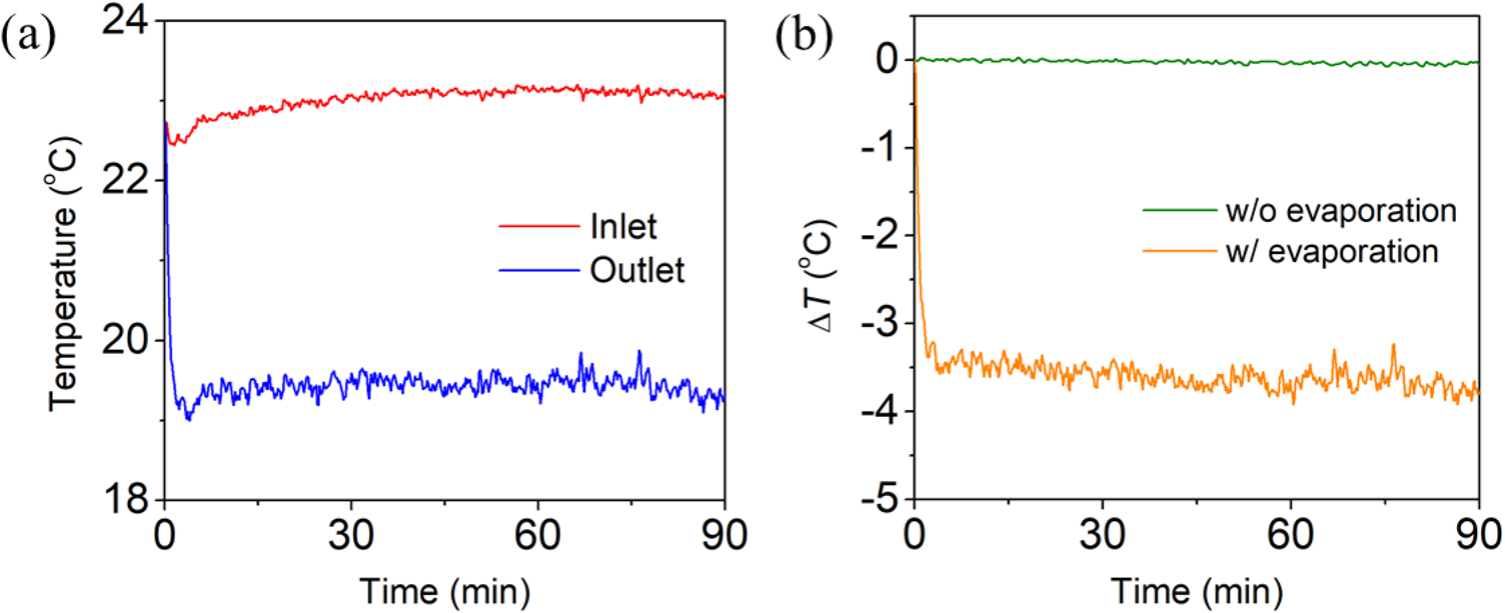
(**a**) Time-dependent changes in the air temperature at the inlet and outlet of the CLS with water evaporation when a 2 m/s wind passed through the CLS. (**b**) The time-dependent differences between the inlet and outlet temperatures of the CLS with and without water evaporation.

A control experiment was conducted to evaluate the evaporative cooling effect of the CLS by measuring the air temperature at the inlet and outlet of the CLS through which 2 m/s wind passed. The experiment was performed in the absence of illumination to avoid radiative effect on temperature measurement. Figure 7a shows that the outlet temperature (19.5 °C) was significantly lower than the inlet temperature (23.1 °C) when a 2 m/s wind passed through the CLS in the presence of water evaporation. In contrast, no temperature difference between the inlet and outlet of the CLS was observed in the absence of water evaporation (not shown here). Figure 7b shows the time-dependent differences between the inlet and outlet temperatures with and without water evaporation. The average temperature difference with water evaporation was 3.6 °C greater than that without water evaporation due to the heat absorption during water evaporation.

The amount of heat absorbed by the CLS under no illumination and 2 m/s wind (Δ*Q*) can be obtained by^56^

3 $$\Delta Q=\frac{{c}_{air}\frac{dm}{dt}\Delta T}{A},$$

where *c*_*air*_ is the specific heat capacity of air (0.700 kJ/kg K), *dm/dt* is the mass flow rate of air passed through the CLS (1.3 × 10^−3^ kg/s), Δ*T* is the air temperature difference between the inlet and outlet of the CLS (3.6 °C), and *A* is the top surface area of the CLS (4.9 × 10^−4^ m^2^). The amount of heat absorbed by the CLS was calculated to be 6.7 kW/m^2^. Since the amount of heat absorbed by the CLS under 1 sun illumination and 2 m/s wind was 9.7 kW/m^2^, the heat absorption by dark evaporation corresponded to ~ 70% of the total heat absorption. Because the 2 m/s wind speed corresponded to only two of the maximum 12 levels according to the Beaufort wind scale^57^, the evaporative cooling effect of the CLS could be greatly improved at higher wind speeds.

## Conclusion

In summary, we have developed a 3D evaporator with an ultrahigh evaporation rate by carbonizing a DLS. The unique porous structure of the DLS consisting of macropores, microchannels, and micro-sized pores remained even after carbonization, and the resulting CLS demonstrated very high performance for solar evaporation during daytime and dark evaporation throughout the day. The water evaporation rate in the absence of illumination and wind was 1.4 kg/m^2^/h, which increased to 14.5 kg/m^2^/h with the presence of a 1-sun illumination and a 2 m/s wind. These results show the highest evaporation rates ever reported. The comparison of the CLS with CMF and PUF revealed that the hierarchically porous structure of the CLS played a key role in the ultrahigh evaporation rate. In particular, the air temperature through which passed the CLS during water evaporation was lower than room temperature because of the absorption of latent heat of vaporization, which demonstrated that CLS can be used to mitigate the UHI effects by promoting evaporative cooling without additional concerns required when trees are used.

## Supplementary Information

Supplementary Information.
