# Supplementary material for: Highly efficient evaporative cooling by all-day water evaporation using hierarchically porous biomass
Source: Sci Rep. 2021 Aug 19;11:16811. doi: 10.1038/s41598-021-96303-w (PMC8376932; doi:10.1038/s41598-021-96303-w)
Supplement: Supplementary file 1 — Supplementary Information. [file 41598_2021_96303_MOESM1_ESM.docx]

Supplementary Information

**Highly Efficient Evaporative Cooling by All-Day Water Evaporation Using Hierarchically Porous Biomass**

Jihun Choi, Hansol Lee, Bokyeong Sohn, Minjae Song, and Sangmin Jeon*

Department of Chemical Engineering, Pohang University of Science and Technology (POSTECH), Pohang, Gyeongbuk, 37673, Republic of Korea


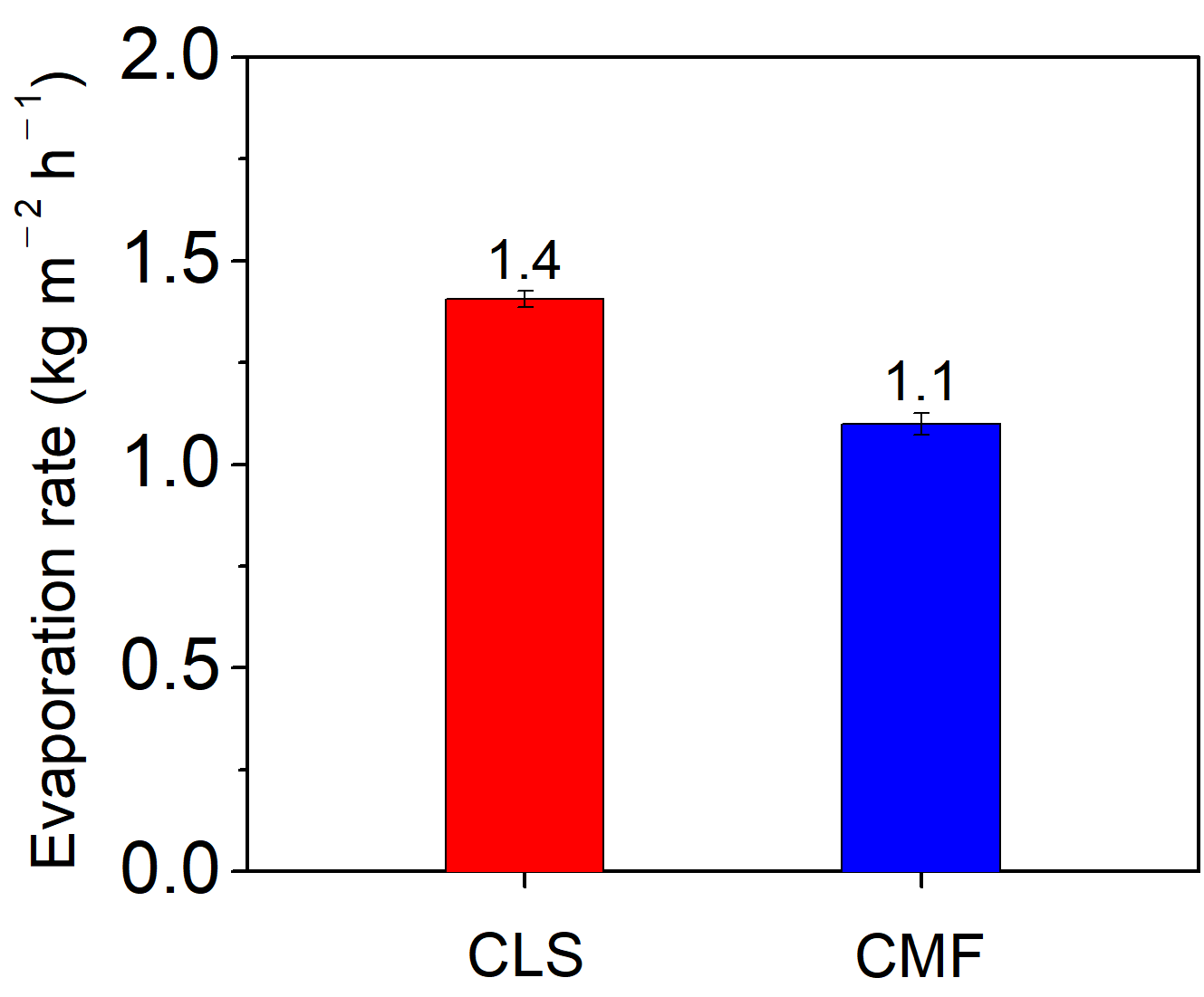


**Fig. S1.** Dark evaporation rates of water with CLS and CMF without illumination and airflow.


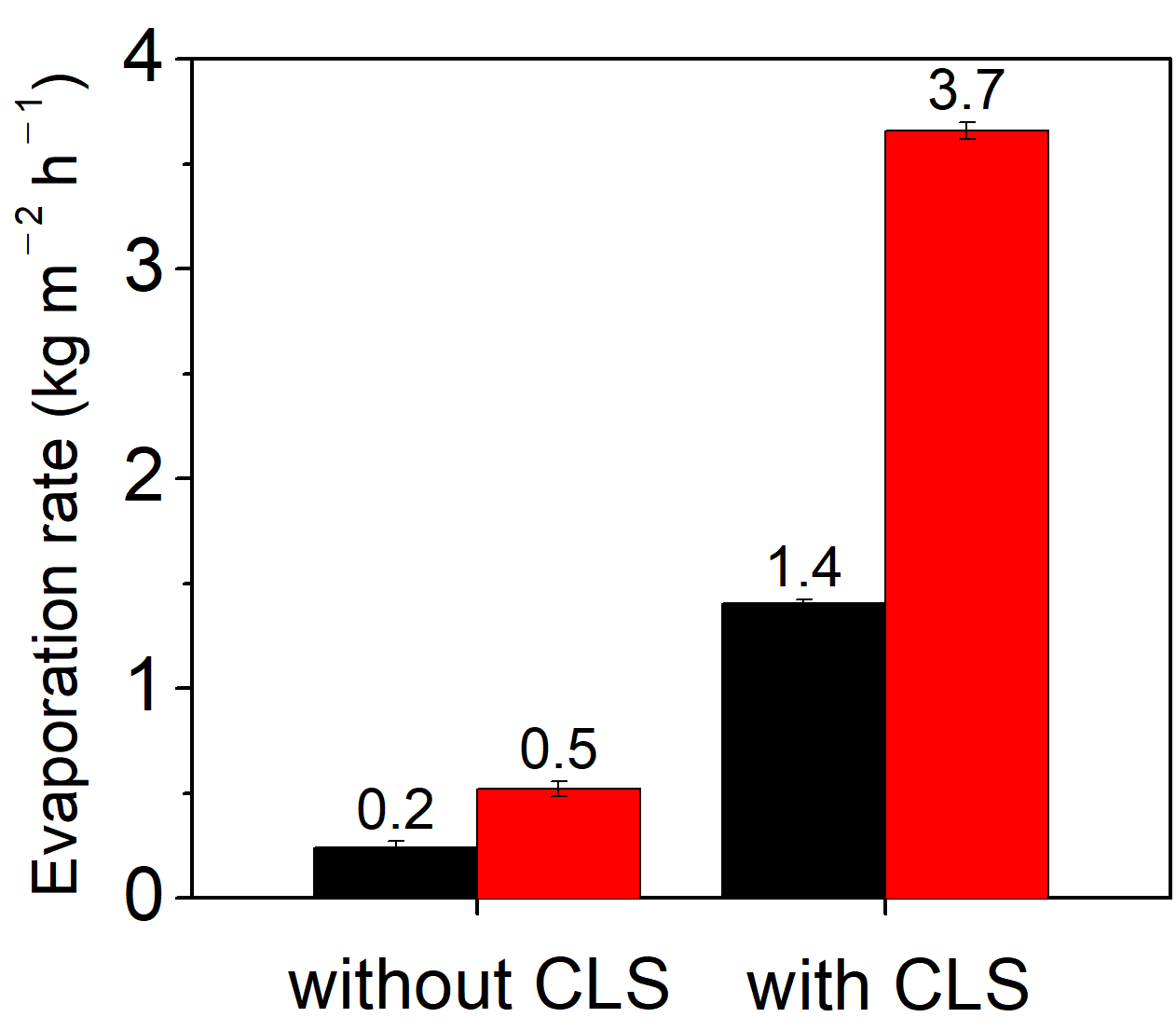


**Fig. S2.** Evaporation rates of water without and with CLS without illumination and wind (black), and under 1-sun illumination and no wind (red).


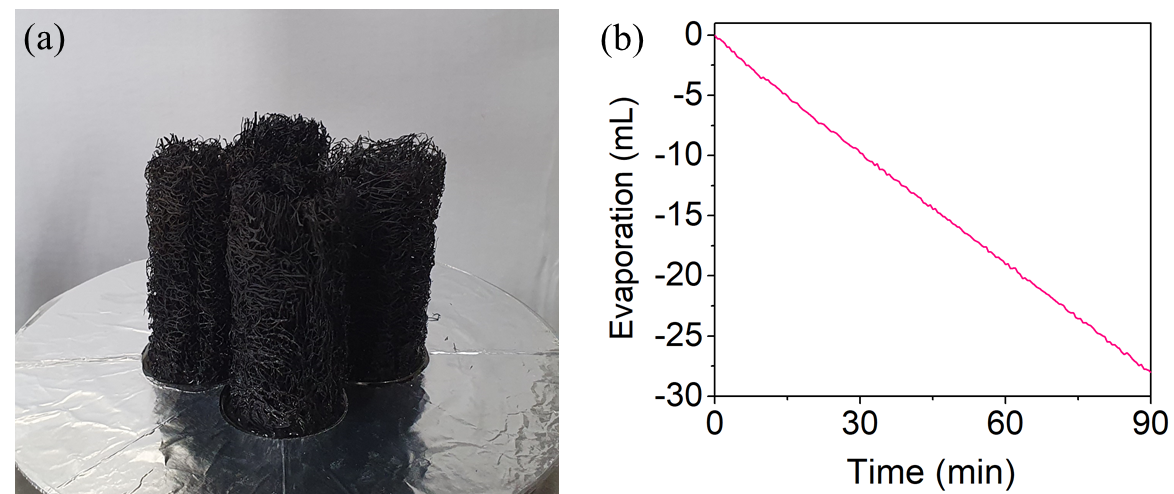


**Fig. S3.** (a) A photo of four CLSs for evaporation experiment. (b) Cumulative evaporation of water with four CLSs under 1-sun illumination and 2 m/s wind.
